# Cytotoxic Effects of Cannabinoids on Human HT-29 Colorectal Adenocarcinoma Cells: Different Mechanisms of THC, CBD, and CB83

**DOI:** 10.3390/ijms21155533

**Published:** 2020-08-01

**Authors:** Daniela Cerretani, Giulia Collodel, Antonella Brizzi, Anna Ida Fiaschi, Andrea Menchiari, Elena Moretti, Laura Moltoni, Lucia Micheli

**Affiliations:** 1 Department of Medical and Surgical Sciences and Neurosciences, University of Siena, 53100 Siena, Italy; daniela.cerretani@unisi.it (D.C.); annaida.fiaschi@unisi.it (A.I.F.); laura.moltoni@unisi.it (L.M.); 2 Department of Molecular and Developmental Medicine, University of Siena, 53100 Siena, Italy; giulia.collodel@unisi.it (G.C.); elena.moretti@unisi.it (E.M.); 3 Department of Biotechnology, Chemistry and Pharmacy, University of Siena, 53100 Siena, Italy; antonella.brizzi@unisi.it; 4 Department of Business and Law, University of Siena, 53100 Siena, Italy; andrea.menchiari@unisi.it

**Keywords:** HT-29 cells, synthetic cannabinoid, Δ^9^-tetrahydrocannabinol, cannabidiol, oxidative stress, apoptosis

## Abstract

In this study, we investigated the effects of exposition to IC_50_ dose for 24 h of a new synthetic cannabinoid (CB83) and of phytocannabinoids Δ9-tetrahydrocannabinol (THC) and cannabidiol (CBD) on HT-29 colorectal carcinoma cells. Cell viability and proliferative activity evaluated using the MTT, lactate dehydrogenase (LDH), and CyQUANT assays showed that cell viability was significantly affected when CB83, THC, and CBD were administered to cells. The results obtained showed that the reduced glutathione/oxidized glutathione ratio was significantly reduced in the cells exposed to CBD and significantly increased in the cells treated with the CB83 when compared to the controls. CBD treatment causes a significant increase in malondialdehyde content. The catalase activity was significantly reduced in HT-29 cells after incubation with CB83, THC, and CBD. The activities of glutathione reductase and glutathione peroxidase were significantly increased in cells exposed to THC and significantly decreased in those treated with CBD. The ascorbic acid content was significantly reduced in cells exposed to CB83, THC, and CBD. The ultrastructural investigation by TEM highlighted a significantly increased percentage of cells apoptotic and necrotic after CB83 exposition. The Annexin V-Propidium Iodide assay showed a significantly increased percentage of cells apoptotic after CB83 exposition and necrotic cells after CBD and THC exposition. Our results proved that only CBD induced oxidative stress in HT-29 colorectal carcinoma cells via CB receptor-independent mechanisms and that CB83 caused a mainly CB2 receptor-mediated antiproliferative effect comparable to 5-Fuorouracil, which is still the mainstay drug in protocols for colorectal cancer.

## 1. Introduction

Cannabinoids obtained from *Cannabis sativa* and their derivatives produce many biological effects, mainly through interactions with specific receptors such as CB1 and CB2, which have been cloned and characterized [1,2]. Moreover, the orphan G protein coupled receptor 55 (GPR55), the transient receptor potential cation channel subfamily V member 1 (TRPV1), and peroxisome proliferator-activated receptors (PPARs) have been reported as possible receptors for endogenous cannabinoids [3,4]. Given the many effects of cannabinoids and the evidence demonstrated by preclinical studies, it is possible to assume a potential use of these substances in the medical field. To date, cannabinoids have been used in the treatment of nausea and vomiting in cancer patients undergoing chemotherapy, but the use of cannabinoids in oncology is likely to be limited, although there is evidence showing that cannabinoids are able to inhibit cell growth in different cancer cell lines [5] and to exert antitumor effects in experimental animal models [6].

**Figure 1:**
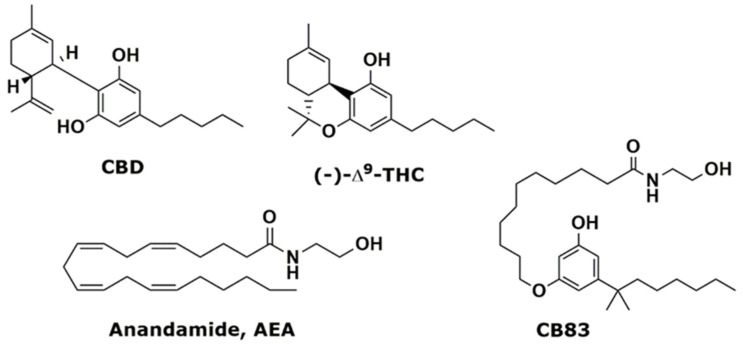
Phytocannabinoids cannabidiol (CBD), Δ9-tetrahydrocannabinol (Δ^9^-THC), endogenous anandamide (AEA), and synthetic cannabinoid CB83.

Through cannabinoid receptor and nonreceptor signaling pathways, cannabinoids show specific cytotoxicity against tumor cells while protecting healthy tissue from apoptosis. Bogdanović et al. [7] investigated the proapoptotic and antiproliferative effects of cannabinoids and associated signaling pathways in different cancer cell lines, and it has been demonstrated that natural and synthetic cannabinoids cause a CB1 and/or CB2 receptor-dependent decrease in the proliferation of breast and intestinal cancer cells [5,8]. Cannabinoids impair tumor progressions at various levels. Their main effect is the induction of cancer cell death by apoptosis and the inhibition of cancer cell proliferation. At least one of those actions has been demonstrated in almost all cancer cell types tested [9]. Cannabinoid treatments affect directly the viability of a great variety of cancer cells via the induction of apoptosis or cell cycle arrest [10,11]. The psychotropic cannabinoid, the Δ^9^-tetrahydrocannabinol (THC, Figure 1), induces apoptosis in a variety of transformed and nontransformed cells, including those of immune origin [10,12]. It was observed that THC treatment induces significant levels of apoptosis in leukemias and lymphocytes in culture, as well as in the murine thymus and spleen [12,13,14], showing that THC may impair T-cell functions through the induction of apoptosis. Moreover, cannabidiol (CBD, Figure 1), a nonpsychotropic cannabinoid, has also been reported to induce apoptosis in several transformed or immortalized cells, including K-ras-thyroid epithelial, C6 glioma, malondhyaldhehyde (MDA)-MB-231 breast carcinoma, HL-60, and Jurkat leukemia cells [5,15,16]. In addition, many evidences suggest that cannabinoids damage tumor angiogenesis and block invasion and metastasis [6,17]. The role of reactive oxygen species (ROS) in regulating apoptosis is supported by many evidences [18], and the production of ROS during apoptosis has been described in various models of apoptotic cell death [19]. Cancer cells seem to possess higher levels of endogenous ROS compared to normal cells, but events that increase ROS levels above a certain threshold seem to be incompatible with the cellular survival. Thus, compounds that increase the ROS level or that impair the cellular antioxidant system will shift the redox balance, inducing cancer cell cytotoxicity [20].

Our previous study in the cannabinoid field led to the development of a new class of synthetic cannabinoid ligands [21,22,23,24,25] chemically characterized by a substituted resorcinol nucleus linked to fatty acid amides. In fact, their structure merges the crucial pharmacophoric requirements for the cannabinoid receptor binding of both THC and anandamide (AEA, Figure 1), the main endogenous cannabinoid, such as a rigid aromatic backbone bearing an alkyl tail and a flexible saturated chain with an amidic head. Among these derivatives, compound CB83 [24], Figure 1, belonging to the 5-(1′,1′-dimethylheptyl)resorcinol class, was selected for its balanced potency (*K*i CB1 = 310 nM and *K*i CB2 = 30 nM) and selectivity.

The aim of this in vitro study was to investigate the effects of the synthetic cannabinoid CB83 and the traditional phytocannabinoids, THC and CBD, on the viability, proliferation, ultrastructure, and apoptosis in human colorectal carcinoma HT-29 cells. We also evaluated the effect of the treatment with cannabinoids on the HT-29 cellular redox state. For this purpose, we determined the parameters of oxidative stress, such as the ratio of reduced glutathione (GSH) to oxidized glutathione (GSSG); the levels of malondhyaldhehyde (MDA), a marker of lipid peroxidation; the antioxidant ascorbic acid (AA); and the cellular antioxidant enzyme activities, such as catalase (CAT), glutathione peroxidase (GPx), and glutathione reductase (GR). Moreover, we compared the CB effects with those observed with the pyrimidine antagonist 5-Fluorouracil (5FU), which is still the mainstay drug in protocols for colorectal cancer.

## 2. Results

### 2.1. CB83, THC, CBD, and 5FU Induce Cytotoxicity and Inhibit the Viability of HT-29 Cells

The results from the MTT assay showed that CB83, THC, CBD, and 5FU were cytotoxic and suppressed the viability of HT-29 cells after 24h, with IC_50_ values of 1.0 ± 0.10 µM, 30.0 ± 1.01 µM, 30.0 ± 3.02 µM, and 34.0 ± 13.89 µM, respectively (Table 1). The HT-29 cells were relatively more sensitive to synthetic cannabinoid CB83, followed by THC, CBD, and 5FU.

In order to verify the cytotoxic activity of CB83, THC, CBD, and 5FU, the viability of HT-29 cells was determined in cellular lysate using the lactate dehydrogenase (LDH) assay. According to the cytotoxic effect found in HT-29 cells, all the substances mentioned above significantly suppressed the viability of the HT-29 cell line after 24 h of treatment, with the maximum effect observed with THC, followed by CB83, 5FU, and CBD, with LDH ratio values of 10.9 ± 0.31, *p* < 0.001; 5.9 ± 1.01, *p* < 0.05; 5.9 ± 0.62, *p* < 0.001; and 5.1 ± 0.72, *p* < 0.05, respectively vs. the control LDH ratio value (3.0 ± 0.07) (Table 1).

The results of the CyQUANT cell proliferation assay, a highly sensitive, fluorescence-based microplate assay, are shown in Table 1. CB83, THC, CBD, and 5FU cause a significant inhibition of proliferation at 24 h (53.1 ± 5.47, *p* < 0.001; 70.9 ± 5.59, *p* < 0.01; 66.8 ± 7.90, *p* < 0.01; and 57.5 ± 6.15, *p* < 0.001, respectively) respective to the control (100.0 ± 10.57).

**Figure 2:**
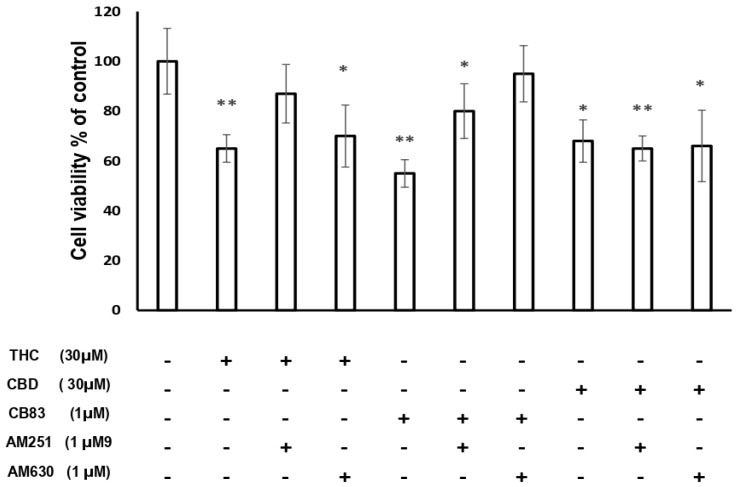
Cytotoxic effects on the cellular viability of HT-29 cells exposed to IC_50_ of THC, CBD, and CB83 in the presence of AM251 (CB1 antagonist) 1μM and AM630 (CB2 antagonist) 1 μM. The results are expressed as % of control. Data are representative of three independent experiments. Data were statistically evaluated. * *p* < 0.05 and ** *p* < 0.01 vs. control.

The results achieved using the incubation with CB1 antagonist AM251 and CB2 antagonist AM630 in HT-29 cells exposed to CBD show that the blockade of CB1 and CB2 receptors did not influence the cytotoxic effect of CBD. The treatment of HT-29 cells with THC in the presence of AM251 did not produce significant effects on the cellular viability compared to the control cells, while using AM630 THC showed a significant reduction of cellular viability (*p* < 0.05 vs. control cells). The treatment of HT-29 cells with CB83 in the presence of CB2 antagonist AM630 did not show significant effects on the cellular viability compared to the control cells, while the cytotoxic effect was maintained in the presence of AM251 (*p* < 0.05 vs. control cells) (Figure 2).

### 2.2. Effects of CB83, THC, CBD, and 5FU on the HT-29 Cellular Redox State

**Figure 3:**
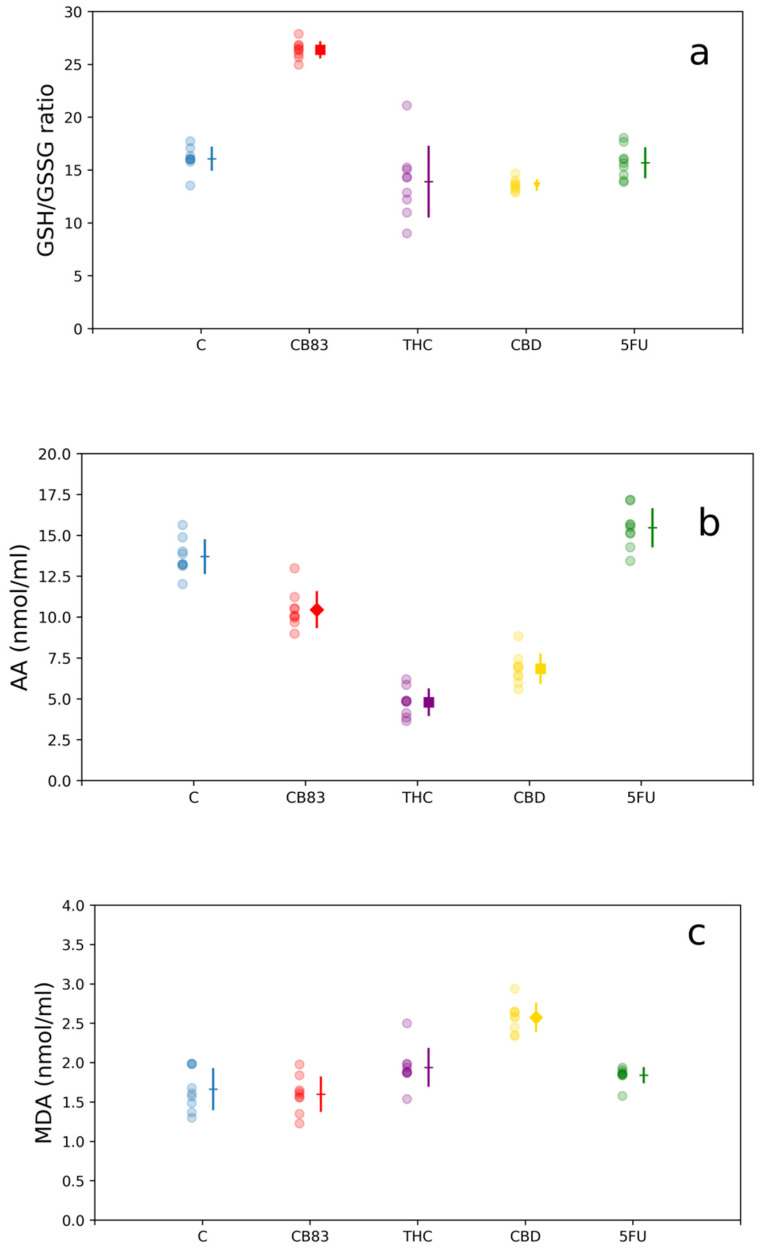
Nonenzymatic antioxidant contents: (**a**) glutathione/oxidized glutathione (GSH/GSSG) ratio, (**b**) ascorbic acid (AA) levels, and (**c**) malondhyaldhehyde (MDA) levels in HT-29 cells exposed to IC50 of CB83, THC, CBD, and 5-Fluorouracil (5FU). Data are representative of three independent experiments. For each treatment, the experimental measurements (left side with circle marker) and mean values ± S.D. (right side) are reported. Mean value markers represent the significance vs. control (C): triangle for *p* < 0.05, diamond for *p* < 0.01, and square for *p* < 0.001; hyphen if dataset is not significant.

In this study we explored the effects of CB83, THC, CBD, and 5FU at the respective IC_50_ doses on the antioxidant cellular defense system in HT-29 cells. For this purpose, the GSH/GSSG ratio; the AA content; the MDA levels as the index of lipid peroxidation; and the activity of antioxidant enzymes CAT, GR, and GPx were determined in cellular lysates after 24 h of incubation. The GSH/GSSG ratio was essentially unchanged in THC and 5FU-treated HT-29 cells, while in the cells exposed to CBD, the GSH/GSSG ratio was significantly reduced (13.7 ± 0.58; *p* < 0.05) respective to the control (16.1 ± 1.14). Conversely, the HT-29 cells treated with the CB83 synthetic cannabinoid showed a significant increase in the GSH/GSSG ratio (26.4 ± 0.83; *p* < 0.001) compared to untreated cells (Figure 3a). The nonenzymatic AA antioxidant content of cells kept for 24 h with CB83, THC, and CBD was significantly reduced (10.5 ± 1.13, 4.8 ± 0.85, and 6.8 ± 0.94 nmol/mL; *p* < 0.01, *p* < 0.001, and *p* < 0.001, respectively) while remaining unchanged in those exposed to 5FU (15.5 ± 1.27 nmol/mL) compared to untreated HT-29 cells (13.7 ± 1.06 nmol/mL) (Figure 3b).

The effects of the treatment of HT-29 with CB83, THC, CBD, and 5FU on MDA levels showed that only the cells exposed to CBD exhibited a significant increase in the MDA content (2.6 ± 0.18 nmol/mL; *p* < 0.01) respective to the untreated cells (1.6 ± 0.27 nmol/mL) to indicate the presence of oxidative damage (Figure 3c).

**Figure 4:**
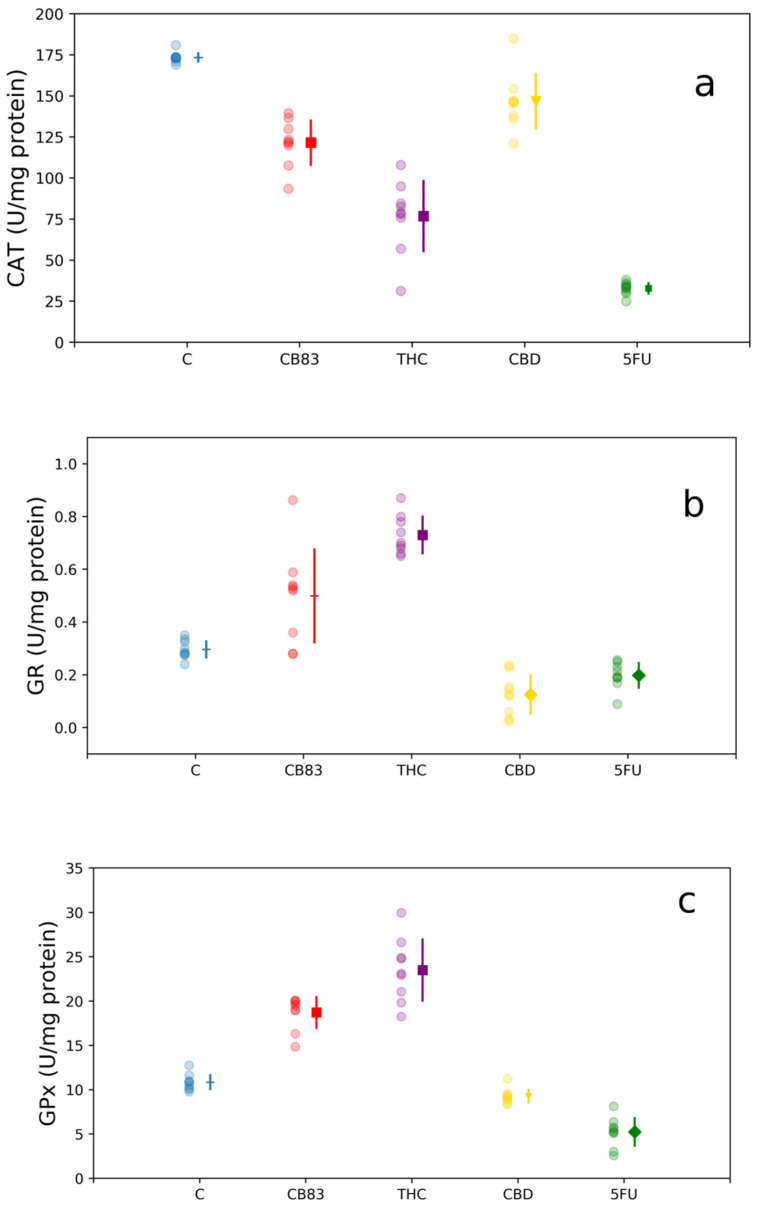
Antioxidant enzymes activity: (**a**) catalase (CAT), (**b**) glutathione reductase (GR), and (**c**) glutathione peroxidase (GPx) in HT-29 cells exposed to IC_50_ of CB83, THC, CBD, and 5FU. Data are representative of three independent experiments. For each treatment, the experimental measurements (left side with circle marker) and mean values ± S.D. (right side) are reported. Mean value markers represent the significance vs. control (C): triangle for *p* < 0.05, diamond for *p* < 0.01, and square for *p* < 0.001; hyphen if dataset is not significant.

The activity of antioxidant enzyme CAT was significantly reduced in HT-29 cells after 24-h incubation with CB83, THC, CBD, and 5FU (121.5 ± 14.03, *p* < 0.001; 76.5 ± 21.92, *p* < 0.001; 146.5 ± 17.26, *p* < 0.05; and 32.9 ± 3.78, *p* < 0.001 U/mg protein, respectively) respective to the control cells (173.1 ± 3.27 U/mg protein) (Figure 4a).

The GR activity appeared to be unaltered in CB83-treated cells (0.5 ± 0.06 U/mg protein), while it showed a significant increase in HT-29 cells exposed to THC (0.7 ± 0.07 U/mg protein, *p* < 0.001). Conversely, the cells treated with CBD and 5FU showed a significant decrease in the enzymatic activity (0.1 ± 0.01 U/mg protein, *p* < 0.01 and 0.2 ± 0.03 U/mg protein, *p* < 0.01, respectively) compared to the control cells (0.3 ± 0.01 U/mg protein) (Figure 4b).

The GPx activity measured in the cells treated with the CB83 proved to be significantly increased (18.7 ± 1.87 U/mg protein, *p* < 0.001), as well as in cells exposed to THC (23.5 ± 3.53 U/mg protein, *p* < 0.001); instead, the HT-29 cells exposed to CBD and 5FU showed a significant decrease in GPx enzymatic activity (9.2 ± 0.79 and 5.2 ± 1.69 U/mg protein, *p* < 0.05 and *p* < 0.01, respectively) respective to the control cells (10.8 ± 0.91 U/mg protein) (Figure 4c).

### 2.3. CB83, THC, CBD, and 5FU Cause Morphological Alterations in HT-29 Cells

#### 2.3.1. AnV/PI Assay

The percentage of intact viable cells was significantly decreased in cells treated with 5FU (*p* < 0.001) and with CB83 (*p* < 0.01) compared to the control cells, while the viability of cells treated with CBD and with THC did not show significant differences, despite an evident increase with that detected in the controls (Table 2). In particular, a significant increase of AnV-positive cells (*p* < 0.001) was shown in CB83 cells with respect to the controls, and a significant increase of necrotic cells (PI-positive) was detected in 5FU (*p* < 0.001), CBD (*p* < 0.01), and THC (*p* < 0.05) cells compared to the controls.

#### 2.3.2. Transmission Electron Microscopy (TEM)

TEM analysis highlighted an increased percentage of apoptotic and necrotic cells in all treated samples respective to the controls (Table 3)—in particular, significant values were detected when the cells were treated with 5FU (*p* < 0.01 and *p* < 0.001, respectively) and with CB83 (*p* < 0.001 and *p* < 0.01, respectively) compared to the control cells.

**Figure 5:**
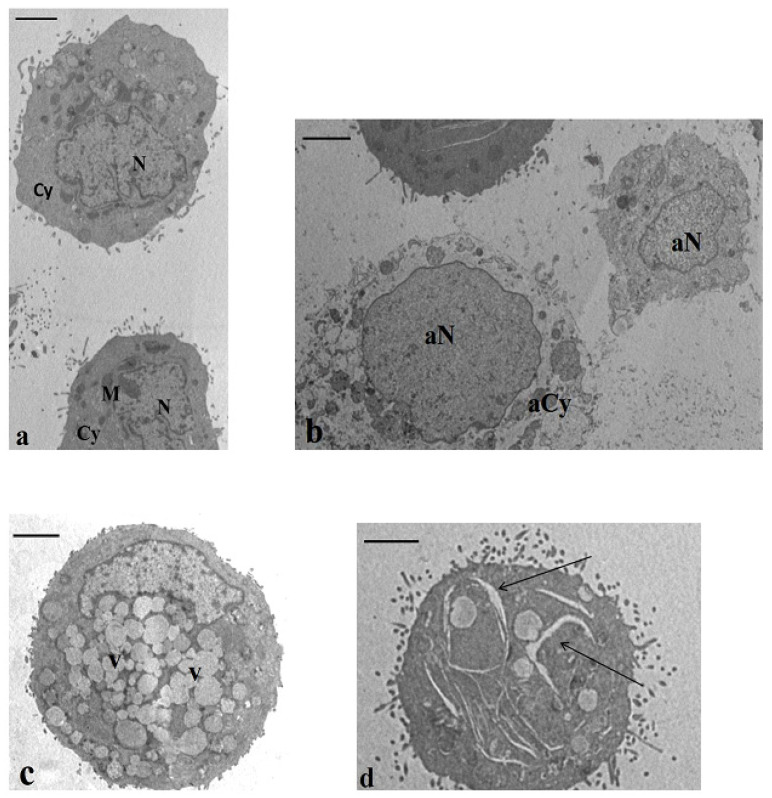
Transmission electron microscopy (TEM) sections of cultured HT-29 cells. (**a**) Baseline conditions. The cells showed normal nuclei (N), regular chromatin texture, and cytoplasm (Cy) containing the typical organelles’ mitochondria (M). Bar 3.5 μm. (**b**–**d**) Incubation with CB83, THC, and CBD. In all samples, a high percentage of cells with necrotic and apoptotic features was described. In (**b**), a necrotic cell is evidenced in an altered chromatin texture (aN) and a cytoplasm devoid of organelles (aCy) and, in (**c**), is represented in an apoptotic cell with a cytoplasm rich in vacuoles (V). The cell showed in (**d**) highlights enlargements in the cytoplasm (arrows). This feature was detected in a percentage of 8–12% in all treated samples. (**b**,**d**) Bar 3.5 μm. (**c**) Bar 3 μm.

Normal cells showed normal chromatin textures and cytoplasm containing rough endoplasmic reticulum, Golgi bodies, and mitochondria (Figure 5a). Necrotic cells (Figure 5b) displayed altered chromatin texture and cytoplasm, deeply impaired and devoid of organelles; the plasma membrane was frequently broken. The apoptotic cell characteristics regarded marginated chromatin and swollen mitochondria; moreover, another ultrastructural peculiar alteration was the presence of a very vacuolated cytoplasm (Figure 5c). In all treated samples, an interesting feature was the presence of a percentage (8–12%) of cells showing the cytoplasm rich in enlargements (Figure 5d). This feature was absent in the control cells.

## 3. Discussion

Cannabinoids act by a modulation of the signaling pathways crucial in the control of cell proliferation and survival, and many in vitro and in vivo experiments have shown that cannabinoids inhibit the proliferation of cancer cells and stimulate autophagy and apoptosis. Here, we studied the effects of cannabinoids THC, CBD, and CB83 on the viability, proliferation, ultrastructure, apoptosis, and cellular redox state of human colorectal carcinoma HT-29 cells. 

MTT results, LDH analyses, and the nucleic acid content determination showed that all the cannabinoids tested and 5FU inhibit cellular proliferation. The HT-29 cells were relatively more sensitive to synthetic cannabinoid CB83, followed by THC, CBD, and 5FU. At the same time, only CBD caused oxidative stress in HT-29 cells. Particularly, in CBD-treated HT-29 cells, the decrease in the GSH/GSSG ratio suggests a redox imbalance towards the appearance of oxidative stress, a condition that did not occur in cells treated with THC, CB83, and 5FU. The evaluation of the activity of cellular antioxidant defense enzymes showed that all cannabinoids tested and 5FU reduced the activity of CAT in HT-29 cells, while the activity of GR was significantly increased in cells treated with THC, reduced in HT-29 cells exposed to CBD and 5FU, and unchanged in the CB83 treatment. The GPx activity was significantly increased in HT-29 cells treated with CB83 and THC, whereas it was reduced in cells exposed to CBD and 5FU. Thus, CBD appears to induce oxidative stress in HT-29 cells, possibly through ROS production, which causes GSH consumption and inhibits CAT, GR, and GPx activities. These data were confirmed by the presence, in CBD-treated cells, of significantly higher MDA levels than in control cells, while they remained substantially unchanged in HT-29 cells exposed to THC, CB83, and 5FU.

The decrease of AA, a nonenzymatic antioxidant, in the cells exposed to cannabinoids can be explained by the increased demand for reducing equivalents necessary to maintain GSH levels through the “sparing” effect of AA on GSSG [26]. In HT-29 cells treated with CBD, the request for reducing equivalents was not satisfied, possibly due to the excess of ROS, and the GSH/GSSG ratio remained below the value of the control, contributing to causing oxidative stress. Massi et al. [15] showed that the CBD-dependent production of ROS was accompanied by a reduction in GSH and GSH-related enzymes. The origin of stress induced by CBD came in part from the mitochondria and led to the activation of multiple caspases involved in the intrinsic and extrinsic pathways of apoptosis. The production of ROS is the most supported hypothesis for the CBD-dependent inhibition of cancer cell aggressiveness in experimental models in cultures, [27,28,29] and Singer et al. [30] observed, for the first time in vivo, that the CBD-dependent generation of ROS is, in part, responsible for the antitumor activity of the cannabinoid.

It is reported that CBD has low affinity for cannabinoid receptors and acts independently of them. In fact, CBD seems to interact with other receptors such as TRPV1, GPR55, or PPARs [3,4]. However, other authors suggested that CBD induces apoptosis in cancer cells partially through the direct or indirect activation of CB2 receptors [5,31]. The results obtained using the incubation with CB1 antagonist AM251 and CB2 antagonist AM630 seem to support the hypothesis that CBD-induced cytotoxicity in HT-29 cells occurs through a CB1 and CB2 receptor-independent mechanism and, possibly, via ROS production, which leads to apoptotic cell death [31,32,33]. Regarding the cytotoxic effects induced in HT-29 by THC and CB83, no signs of oxidative stress were evident in the cells treated with these cannabinoids. Furthermore, THC in the presence of AM251 did not produce significant effects on the cellular viability compared to the control cells; while using AM630, THC showed a significant reduction of the cellular viability to demonstrate that it is an agonist of the CB1 receptor, with less efficacy at the CB2 ones [34].

This result is in-line with what was observed by other authors [35], even if it was reported that antitumor activities of THC were associated with both cannabinoid CB1 and CB2 receptors [36,37,38]. Otherwise, the treatment of HT-29 cells with CB83 in the presence of CB2 antagonist AM630 does not show significant effects on the cellular viability compared to the control cells, while the cytotoxic effect is maintained in the presence of AM251, the selective CB1 receptor antagonist. The synthetic cannabinoid CB83 has been shown to have a higher CB2 affinity (*K*i = 30 nM), rather than for CB1 receptors (*K*i = 310 nM). These data seem to confirm that the cytotoxic effect of CB83 is mainly mediated by CB2 receptors. The inhibition of the cancer cell proliferation and induction of apoptosis in cancer cells by CB1 and CB2 activation is described, and the most upstream key molecule that can initiate death signals appears to be ceramide [39]. The increases of ceramide production via a mechanism that involves tumor necrosis factor-alpha (TNF-α) [34] induces an activation of the endoplasmic reticulum stress-related signaling pathway, which, finally, leads to the activation of the intrinsic apoptosis pathway [31].

The analysis by the Annexin V-Propidium Iodide assay and TEM confirmed that all the tested cannabinoids increased the percentages of the apoptotic and necrotic cells, even if only the synthetic cannabinoid CB83 determined a significant increase of these pathologies. Specifically, this compound resulted as effective as 5FU. These results agree with the data obtained by the MTT test.

Probably through cannabinoid receptors and nonreceptor signaling pathways, cannabinoids showed specific cytotoxicity against tumor cells [7]. By TEM analysis, this cytotoxic effect, particularly evident in samples incubated with CB83, was highlighted by the significant increase of cells with marginated chromatin, swollen mitochondria, and vacuolated cytoplasm or disrupted chromatin and broken plasma membrane, typical features, respectively, of apoptosis and necrosis.

The cannabinoid ligands have been shown to sensitize cancer cells and, synergistically, may interact with members of the TNF receptor, thus suggesting that the combination of cannabinoids with death receptor ligands induces additive or synergistic tumor cell death [40]. Recently, it has been reported that different compounds induced cell death by necroptotic and apoptotic mechanisms in cancer cells, with a concomitant mitochondrial metabolism failure that triggers lower production of ATP and ROS overproduction [41].

## 4. Materials and Methods

### 4.1. Chemicals

All reagents were purchased from Sigma-Aldrich (St. Louis, MO, USA). All solutions were prepared with deionized water of resistivity no less than 18.2 MΩ·cm^–1^ (Milli-Q Ultrapure Water System, Merck KGaA, Darmstadt, Germany).

### 4.2. Cell Cultures

Human colorectal carcinoma HT-29 cells were purchased from ATCC (American Type Culture Collection, Manassas, VA, USA), and they were cultured in Dulbecco’s modified Eagle’s medium (MEM) + GlutaMAX™-1 supplemented with 10% fetal bovine serum (FBS) and 1% penicillin-streptomycin (Gibco/Invitrogen, Carlsbad, CA, USA). The cells were grown in T-75 flasks at 37 °C in a humidified atmosphere with 5% CO_2_ in the air, and the culture medium was replaced every 2 to 3 days, and passages were performed 1 to 2 days per week.

### 4.3. Cell Viability Assays

#### 4.3.1. Assay for Cytotoxicity (MTT Assay)

To explore the antiproliferative effect on the HT-29 cell line, the cells were exposed to CB83, THC, CBD, and 5FU for 24 h, and the effect on the cell viability was determined using the MTT assay.

The HT-29 cells were preincubated in a 96-well plate at a density of 1.0 × 10^4^ cells/well for 24 h; cells were treated with CB83, THC, CBD, and 5FU at different concentrations (range from 0.1 mM to 0.1 nM) to determine the IC_50_ values. After incubation for 24 h, the MTT reagent (5 mg/mL) was added to each well, and the plate was incubated for an additional 4 h at 37 °C. At the end of incubation, the media were removed, and the intracellular formazan product was dissolved in 100 μL of isopropyl alcohol. The absorbance of each well was measured at 540 nm using an ELISA reader (iMarkTM; Bio-Rad Laboratories, Inc., Hercules CA, USA), and the MTT reduction rate was calculated by setting each of the control survivals equal to 100%.

In order to investigate the role of the CB receptors, the MTT assay was again performed with specific antagonists of the CB1 and CB2 receptors. The cells were pretreated for 30 min with CB1 and CB2 antagonists AM251 (1 μM) and AM630 (1 μM), respectively, before the addition of CB83, THC, and CBD.

#### 4.3.2. Lactate Dehydrogenase (LDH) Determination

To evaluate the cellular viability in HT-29 cells, the cells were plated at an initial density of 2 × 10^5^ cells/well in 6-well plates, and they were allowed to attach overnight. Afterwards, the medium was removed by aspiration, and the cells were exposed to the IC_50_ values of CB83, THC, CBD, and 5FU for 24 h. Cells lysate and culture media samples were recovered to the assay LDH activity. LDH catalyzed the conversion of pyruvate to L-lactate, while the reduced nicotinamide adenine dinucleotide (NADH) was oxidized. The rate of oxidation, which is directly proportional to the LDH activity [42], was monitored by measuring the decrease in absorbance at 340 nm using an UV-Vis Spectrophotometer (Lambda 35, Perkin Elmer, Norwalk, CT, USA). Total LDH activity was evaluated by calculation ΔA/min in lysate and media samples. Viability was expressed as the LDH ratio (LDH media/LDH lysate). 

#### 4.3.3. Cell Proliferation Assay

The cell viability was determined by using the CyQUANT cell proliferation assay kit, which measures the total nucleic acid content. The cells were plated in 6-well plates at a seeding density of 2 × 10^5^ in 3 mL of culture medium containing 1% FBS. Then, the cells were treated with CB83, THC, CBD, and 5FU for 24 h at the respective IC_50_ doses. The cells were harvested and were seeded (5000 cells/well) into a 96-well microplate in 200 µL of culture medium containing 1% FBS for 4 h. The medium was aspirated, and the plates frozen at −80 °C until used. The plates were thawed at room temperature and processed according to the manufacturer’s instructions. The analysis was carried out using a VICTOR Multilabel plate reader (excitation 485 nm/emission 520 nm; Perkin Elmer Victor 3V, Waltham, MA, USA).

### 4.4. Cellular Redox Systems Evaluation

HT-29 cells were exposed to CB83, THC, CBD, and 5FU to evaluate a panel of factors involved in the oxidative stress response.

The cells were plated at an initial density of 2 × 10^5^ cells/well in 6-well plates, allowing them to attach overnight. Afterwards, the medium was removed by aspiration, and the cells were treated to respective doses IC_50_ of CB83, THC, CBD, and 5FU for 24 h. Then, cells were detached and centrifuged at 1500× *g* for 2 min. The pellets were resuspended in 1 mL of phosphate-buffered saline (PBS) and exposed to three cycles of freeze-thaw, freezing at −80 °C in the freezer.

The supernatants of cells destroyed by freezing and thawing were aliquoted and processed as described below in the respective methods.

#### 4.4.1. Glutathione Oxidized and Reduced

An aliquot of cell lysates was added to an equal volume of 10% metaphosphoric acid and centrifuged at low speed (2000× *g*) for 10 min at 0 °C. The supernatant was removed and stored at −80 °C until use. Total GSH and GSSG levels were quantified in the supernatant using a a micro-assay procedure [43] based on an enzymatic method with the reading at 415 nm. Results were expressed in nmol/mg protein.

#### 4.4.2. Proteins Assay

Protein concentrations were determined by the method of Lowry et al. [44], and the calibration curves were prepared with dry bovine serum albumin.

#### 4.4.3. AA Assay

AA levels were measured in the aliquot of cell lysates acidified with metaphosphoric acid using an HPLC method, as described by Ross [45], with minor modifications. The supernatants were filtered (Anotop 0.2 μm, Merck), and 20 μL were injected into a high-performance liquid chromatography (HPLC) column. The AA was quantified by UV reverse-phase HPLC using a Waters 600 E System Controller HPLC (Milford, MA, USA) equipped with a Waters Dual λ 2487 UV detector (Milford, MA, USA) set at 262 nm. A 5-µm ultrasphere ODS column (Beckman, San Ramon, CA, USA) was used with the acetonitrile-water (49/51, *v*/*v*) as the mobile phase at the flow rate of 0.8 mL/min. The AA concentrations (nmol/mL) were calculated by peak areas, determined using an Agilent 3395 integrator (Agilent Technologies, Santa Clara, CA, USA).

#### 4.4.4. Malondialdehyde Assessment

The extent of lipid peroxidation in cell lysates was estimated by calculating the MDA levels according the method of Shara et al. [46], with minor modifications. To prevent artifact oxidations of polyunsaturated free fatty acids, immediately after the freezing and thawing, an aliquot of 0.2 mL was added to 0.2 mL of tris-HCl 0.04 M and acetonitrile containing 0.1% butylated hydroxytoluene. After centrifugation at 2000× *g* for 10 min at 0 °C, the supernatant was frozen at −80 °C until use.

At the time of analysis, the supernatant was derivatized with 2,4-dinitrophenylhydrazine and immediately stirred and extracted with 5 mL of pentane; finally, the samples were dried using nitrogen. A calibration curve with concentrations of MDA in the range from 0.5 nmol/mL to 10 nmol/mL was used.

The MDA hydrazone was quantified by isocratic HPLC using a Waters 600 E System Controller HPLC (Milford, MA, USA) equipped with a Waters Dual λ 2487 UV detector (Milford, MA, USA) set at 307 nm. A 5-µm ultrasphere ODS column C18 (Beckman, San Ramon, CA, USA) was used to separate the hydrazone derivative at the flow rate of 0.8 mL/min with the acetonitrile (45%)-HCl 0.01 N (55%) as the mobile phase. The MDA concentrations (nmol/mL) were calculated by peak areas determined using an Agilent 3395 integrator (Agilent Technologies, Santa Clara, CA, USA).

#### 4.4.5. Catalase Activity

An aliquot of cell lysates was centrifuged at 4000× *g* for 15 min at 4 °C, and the supernatants were frozen at −80 °C until use. To determine the CAT, a micro-assay procedure was used [47].

This method is based on the reaction of the CAT with methanol in the presence of an optimal concentration of hydrogen peroxide. The formaldehyde production was measured spectrophotometrically at 540 nm with 4-amino-3-hydrazino-5-mercapto-1,2,4-triazole (Purpald) as a chromogen. One unit of CAT activity is defined as the amount of enzyme that will cause the formation of 1 nmol of formaldehyde per minute at 25 °C. The results were expressed as U/mg protein.

#### 4.4.6. Glutathione Reductase Activity

For the evaluation of the GR activity, an aliquot of cell lysates was diluted (1:1) in cold 0.25-M sucrose in 0.1-M phosphate buffer, pH 7.4, and centrifuged at 40,000× *g* for 20 min at 4 °C. The supernatants were stored at −80 °C until analyzed. The method is based on the increase in absorbance at 415 nm when 5,5′-dithiobis(2-nitrobenzoic acid) is reduced by GSH generated from an excess of GSSG [48].

Samples were prepared in 96-well plates, and absorbance was measured every 30 s for 3 min with a programmable microplate reader. The rate of increase in absorbance was directly proportional to the amount of GR in the sample. The results were expressed as U/mg protein.

#### 4.4.7. Glutathione Peroxidase Assay

The cell lysates were treated using the same procedure as described for GR. The GPx activity is quantitated by measuring the change in absorbance at 340 nm caused by the oxidation of NADPH [49]. One unit of GPx activity is defined as the amount of enzyme that oxidizes 1 µmol of NADPH at 37 °C per minute. Enzyme activity was expressed as U/mg protein.

### 4.5. Morphological Studies

Apoptosis was initially induced by the incubation of cells 2 × 10^4^ cells/well in complete growing medium for 24 h with the respective IC_50_ doses of CB83, THC, CBD, and 5FU.

#### 4.5.1. Transmission Electron Microscopy (TEM)

Samples were fixed in cold Karnovsky fixative and maintained at 4 °C for 2 h. Fixed cells were washed in 0.1-M cacodylate buffer (pH 7.2) for 12 h, post-fixed in 1% buffered osmium tetroxide for 1 h at 4 °C, then dehydrated and embedded in Epon Araldite. Ultra-thin sections were cut with a Supernova ultramicrotome (Reickert Jung, Vienna, Austria), mounted on copper grids, stained with uranyl acetate and lead citrate, and then observed and photographed with a Philips CM12 transmission electron microscope (TEM; Philips Scientifics, Eindhoven, The Netherlands and Centro di Microscopie Elettroniche “Laura Bonzi”, ICCOM, Consiglio Nazionale delle Ricerche—CNR, Via Madonna del Piano, 10 Firenze, Italy).

One-hundred ultra-thin cell sections were analyzed from each sample. Major submicroscopic characteristics were recorded by two highly trained examiners who were blind to the experiment applying the same evaluation criteria. Cell conditions (normal, apoptosis, and necrosis) were defined by typical ultrastructural characteristics. Marginated chromatin, cytoplasmatic translucent vacuoles, and swollen and badly assembled mitochondria were the typical ultrastructural markers of apoptosis, whereas cells with a broken plasma membrane and nuclei with disrupted chromatin were affected by necrosis. TEM evaluation was carried out in three different experiments.

#### 4.5.2. Annexin V/Propidium Iodide Assay

The detection of phosphatidylserine (PS) externalization was performed with the Vybrant Apoptosis Assay kit (Invitrogen Ltd., Paisley, UK) made up of Annexin (An) V-fluorescein isothiocyanate (FITC) and propidium iodide (PI), which are able to differentiate viable from apoptotic/necrotic cells. The samples were washed with PBS, centrifuged, and suspended in Annexin-binding buffer (ABB) to obtain a cell density of ~1 × 10^6^. Following the manufacturer’s instructions, 10 µL of conjugated-FITC AnV and 1 µL of PI (100 µg/mL) working solution to each cell suspension were added. Cells were incubated in the dark for 15 min at 37 °C. After a careful wash with ABB, a drop of cell suspension was smeared on each glass slide. Slides were mounted in glycerol containing 5% n-propylgallate. Observations were made with a Leitz Aristoplan (Leica, Wetzlar, Germany) light microscope equipped with a fluorescence apparatus. A total of 300 cells from each sample were counted. By staining cells with FITC-AnV (AnV, green fluorescence) and, simultaneously, with the nonvital dye (PI, red fluorescence), it is possible to recognize intact cells (AnV-negative and PI-negative), early-apoptotic cells with PS externalization (AnV-positive and PI-negative), cells with PS externalization and damaged membranes (AnV-positive and PI-positive), and necrotic cells (AnV-negative and PI-positive). The results were expressed as the percentages of apoptotic cells (green).

### 4.6. Statistical Analysis

All experiments were independently repeated three times, with multiple replicates within each run. The data represented the mean of three independent experiments in triplicate and were expressed as means ± SD. Statistical analysis was performed using SPSS v.19 Chicago: SPSS Inc. (Chicago, IL, USA).

The IC_50_ value was defined as the concentration causing proliferation inhibition by 50% compared to the control. The IC_50_ values are given as mean values ± SD and were calculated according to the method of Litchfield and Wilcoxon [50].

Data was tested for normality using a Kolomogorov-Smirnov Test.

Statistical comparisons were made by one-way ANOVA or Kruskal–Wallis test.

Tukey’s post-hoc test was used under homoscedasticity conditions. Games-Howell post-hoc test was used on violations of the assumption of homoscedasticity. Dunn’s multiple comparisons test for Kruskal–Wallis tests. The values were considered significantly different when *p* ≤ 0.05.

## 5. Conclusions

In summary, we found that CB83, a new synthetic cannabinoid characterized by an alkylresorcinol nucleus, has an effect comparable to 5FU, which is still the mainstay drug in protocols for colorectal cancer. CB83 appears to produce its effects mainly through CB2 receptors and is more effective than CBD and THC in the induction of apoptosis and necrosis in HT-29 cells. Furthermore, in this work we observed, like other authors, the cytotoxic effect of CBD on HT-29 cells appears to be independent of the activation of CB1 and CB2 receptors and, instead, to involve the production of ROS and, consequently, oxidative stress, which leads cells to apoptosis and necrosis. Therefore, a combined treatment that exploits the synergistic cytotoxic effects of CB83 and CBD on colon cancer cells could represent an interesting new strategy in colon cancer therapy. Further studies will be needed to evaluate the efficacy and toxicity of CB83 and the CB83 + CBD association in animal models.

## Figures and Tables

**Table 1:** Results from the MTT, lactate dehydrogenase (LDH), and CyQUANT assays.

Treatment	MTT IC_50_ (μM)	LDH ratio	CyQUANT %
C	-	3.0 ± 0.07	100.0 ± 10.57
CB83	1.0 ± 0.10	5.9 ± 1.01 *	53.1 ± 5.47 ***
THC	30.0 ±1.01 ^##^	10.9 ± 0.31 ***	70.9 ± 5.59 **
CBD	30.0 ± 3.02 ^##^	5.1 ± 0.72 *	66.8 ± 7.90 **
5FU	34.0 ± 13.89 ^#^	5.9 ± 0.62 ***	57.5± 6.15 ***

Data are representative of three independent experiments and are presented as mean ± S.D. Data were statistically evaluated. ^#^ *p* < 0.05 and ^##^ *p* < 0.01 vs. CB83 and * *p* < 0.05, ** *p* < 0.01, and *** *p* < 0.001 vs. control (C). THC: tetrahydrocannabinol, CBD: cannabidiol, and 5FU: 5-Fluorouracil.

**Table 2:** Results from screening with the Annexin V(AnV)-fluorescein isothiocyanate (FITC) and propidium iodide (PI) assay. Intact cells appeared unstained (AnV- or PI-), apoptotic cells with phosphatidylserine (PS) externalization were green stained with FITC-Annexin (AnV+ PI-), and necrotic cells were red stained (AnV- PI+ or AnV+ PI+) with the broken plasma membrane.

	Intact % AnV-PI-	Apoptosis % AnV+PI-	Necrosis % AnV+PI+
C	84.0 ± 1.01	11.0 ± 1.15	3.7 ± 1.15
5FU	38.7 ± 1.53 ***	31.3 ± 1.53	32.3 ± 2.09 ***
CB83	43.3 ± 0.58 **	37.3 ± 1.15 ***	19.3 ± 1.53
CBD	53.0 ± 0.10	20.6 ± 1,16	27.0 ± 1.05 **
THC	56.3 ± 2.52	19.6 ± 2.08	25.0 ± 1.73 *

Data are representative of three independent experiments and are presented as mean ± S.D. Data were statistically evaluated. * *p* < 0.05, ** *p* < 0.01, and *** *p* < 0.001 vs. control (C).

**Table 3:** Results from screening with a transmission electron microscopy (TEM analysis. One hundred cell sections were analyzed for each sample. A cell was considered apoptotic when marginated chromatin, translucent vacuoles, and swollen and badly assembled mitochondria were detected. A cell was considered necrotic when broken plasma membrane and disrupted chromatin were observed.

	Apoptosis %	Necrosis %
C	12.7 ± 2.08	3.3 ± 1.53
5FU	27.3 ± 1.53 **	28.3 ± 1.54 ***
CB83	35.0 ± 2.01 ***	26.0 ± 2.05 **
CBD	22.0 ± 1.10	22.7 ± 2.52
THC	20.0 ± 0.01	20.0 ± 1.73

Data are representative of three independent experiments and are presented as mean ±S.D. Data were statistically evaluated ** *p* < 0.01, and *** *p* < 0.001.
